# Pathological Findings in Eurasian Otters (*Lutra lutra*) Found Dead between 2015−2020 in Schleswig-Holstein, Germany

**DOI:** 10.3390/ani12010059

**Published:** 2021-12-28

**Authors:** Simon Rohner, Peter Wohlsein, Ellen Prenger-Berninghoff, Christa Ewers, Patrick Waindok, Christina Strube, Christine Baechlein, Paul Becher, Dunja Wilmes, Volker Rickerts, Ursula Siebert

**Affiliations:** 1 Institute for Terrestrial and Aquatic Wildlife Research, University of Veterinary Medicine Hannover, Foundation, 30559 Hannover, Germany; simon.rohner@tiho-hannover.de; 2 Department of Pathology, University of Veterinary Medicine Hannover, Foundation, 30559 Hannover, Germany; peter.wohlsein@tiho-hannover.de; 3 Institute of Hygiene and Infectious Diseases of Animals, Justus Liebig University Giessen, 35390 Giessen, Germany; ellen.prenger-berninghoff@vetmed.uni-giessen.de (E.P.-B.); christa.ewers@vetmed.uni-giessen.de (C.E.); 4 Centre for Infection Medicine, Institute for Parasitology, University of Veterinary Medicine Hannover, Foundation, 30559 Hannover, Germany; patrick.waindok@freenet.de (P.W.); christina.strube@tiho-hannover.de (C.S.); 5 Institute of Virology, University of Veterinary Medicine Hannover, Foundation, 30559 Hannover, Germany; christine.baechlein@laves.niedersachsen.de (C.B.); paul.becher@tiho-hannover.de (P.B.); 6 Lower Saxony State Office for Consumer Protection and Food Safety (LAVES), Food and Veterinary Institute Braunschweig/Hannover, 30173 Hannover, Germany; 7 Robert Koch Institute, 13353 Berlin, Germany; WilmesD@rki.de (D.W.); RickertsV@rki.de (V.R.)

**Keywords:** Eurasian otter, population health, monitoring, dissection, infectious diseases, roadkill

## Abstract

**Simple Summary:**

Working with protected and elusive wildlife species remains a challenge when it comes to population health investigations. Specimens found dead, such as road-killed animals, can therefore serve as a valuable source to address various questions concerning the population level. We herein describe pathological findings in Eurasian otters found dead in Schleswig-Holstein, Germany, which were obtained using a special otter dissection protocol. To actually monitor population trends and the health of otters, the continuation of post-mortem investigations is essential.

**Abstract:**

In times of massive biodiversity loss and ongoing environmental crises, it is extremely important to ensure long-term conservation efforts of threatened species like Eurasian otters (*Lutra lutra*). To gain insights into the status of Northern Germany’s otter population, 92 otters found dead in Schleswig-Holstein between 2015−2020 were collected and underwent detailed dissection with the aim to establish a monitoring program for this population. Examinations followed a protocol especially designed for otters, including various biological data assessments and extended sampling. The finding sites showed a clear concentration in the Continental region. Seasonal concentration differed among the years, yet peaks were seen from fall to winter. Overall, more males than females were found, although this differed among the years. The majority of otters that could be aged were between 1–3 years. Placental scars and pregnancy were recorded in only few females. Nutritional status was good in most cases. Infectious diseases found included *Vagococcus lutrae*, *Toxoplasma gondii*, and *Emmonsia* spp. A major cause of death was roadkill. Known sample bias in studies focusing on roadkill was considered in the finding interpretation. Continuation of the population health investigations is mandatory to analyze potential trends and to establish an actual monitoring program for Eurasian otters in Schleswig-Holstein.

## 1. Introduction

After huge declines in the past, the Eurasian otter (*Lutra lutra*) (Linnaeus, 1758) population is recolonizing former habitats in Western Europe, including Germany [1,2,3]. This includes the northernmost German federal state Schleswig-Holstein, where otters are detected area-wide nowadays, and which functions as an important bridge between German and Danish populations [4,5,6]. *Lutra lutra* remains a species that is strictly protected by national [7] and international law [8]. The International Union for the Conservation of Nature (IUCN) lists Eurasian otters as “near threatened” globally [9], whereas this differs among countries and also federal states in Germany [3,10,11]. During the last decades, there has been a rapid increase in the number of otters found dead in Schleswig-Holstein, Germany (A. Drews, pers. communication), together with sightings and population surveys [12] indicating a growing population and demanding a better understanding of the reestablishment of otters. To allow for successful long-term population management, detailed post-mortem investigations are essential for elusive and protected species like *Lutra lutra* [13]. Standardized necropsies of otters found dead can serve as a valuable diagnostic tool to assess the health status of populations [14,15,16,17]. As seen in other studies, otters were infected with dolphin morbillivirus in Italy [18], unspecified distemper virus in Denmark and Germany [19], or canine and feline parvovirus in Southern Italy [20]. The role of otters in the current SARS-CoV-2 pandemic still remains unclear, whereas other mustelids are highly susceptible to the virus [21,22]. Bacteria, such as certain streptococci [15,23] or zoonotic *Vagococcus lutrae* [24], also affect otter populations. Pneumonia due to fungal agents, such as *Emmonsia* spp., leading to clinical adiaspiromycosis, reportedly occur among otters [25,26]. Other relevant infectious agents include ectoparasites, such as ticks [27,28], and endoparasites like *Sarcocystis lutrae* [29,30], *Isthmiophora melis* [31,32], *Corynosoma* spp. [33,34], or *Toxoplasma gondii* [35,36]. Still, there is certain bias that needs to be taken into account, especially when using mainly roadkill [17]. The latest published otter post-mortem data from Germany are restricted to the population in eastern federal states [17,32,37]. This study aimed to investigate the health status and causes of death of the otter population in Schleswig-Holstein in order to set baselines for an actual monitoring program, including assessment of infectious agents, age determination, sex and reproductive activity.

## 2. Materials and Methods

### 2.1. Network and Carcass Collection

In 2019, a program to investigate Eurasian otter population health was established in Schleswig-Holstein and was funded for two years by the Ministry for Energy Transition, Agriculture, Environment, Nature, and Digitalization (MELUND), with the aim to collect baseline information for future monitoring. All otter carcasses found dead in 2019 and 2020 were collected by a network involving stakeholders, street maintenance depots, hunters, conservationists, and other trained people interested in otters. Another 27 frozen otters from Schleswig-Holstein, which had been collected opportunistically in the previous years, 2015 to 2018, were provided by the Zoological Institute, Christian-Albrechts University, Kiel, Germany (CAU). All animals were processed at the Institute for Terrestrial and Aquatic Wildlife Research (ITAW) in Büsum, Germany. Most carcasses were stored at −20 °C until dissection, and some were dissected in fresh condition. Some individuals were deskinned by taxidermists before dissection, which limited data collection and sampling.

### 2.2. Dissection

Following Siebert et al. [38], as well as international otter examination schemes available online, a dissection protocol for otters was designed in 2019 (Figure S1: Dissection Protocol-Otter). Nineteen of the twenty-seven otters provided by Kiel University from previous years had been investigated before 2019 using a less specified protocol, which limited the comparability of these specific individuals. Except those, all otters were necropsied by the same expert veterinarian. Depending on the decomposition level (1 = fresh; 2 = good; 3 = moderate; 4 = progressed decomposition; 5 = severe decomposition) and if the animal had been previously deskinned, data assessment and sampling varied. In general, the animals were weighed, and various measurements of the body length, girth, etc., of the otters were taken (see Supplementary Materials). Biometric data such as the length and weight were always taken after thawing. Careful palpation allowed for the detection of bone fractures. Nutritional state (good, moderate, or poor) was judged based on the occurrence of subcutaneous fat depots, especially in the tail radix region, of retroperitoneal fat depots in the abdomen, and the development of certain muscle groups along the spine. The reproduction status was assessed, checking for prominent mammary glands, placental scars, and fetuses in females [14,39]. Placental scars indicate former pregnancy until three months post partum, whereas direct comparison regarding the number of cubs may be limited due to pre- and perinatal loss [40].

### 2.3. Histology

All of the organ systems were examined, and various tissue samples were taken for histopathological analysis, in accordance with Siebert et al. [41]. Only moderate to severe findings based on a semiquantitative estimation were taken into consideration. Organs routinely assessed histologically included tongue, tonsils, retropharyngeal lymph node, trachea with larynx, esophagus, thyroid glands, thymus, diaphragm, lungs, pulmonal lymph node, heart, aorta, liver with gall bladder, pancreas, stomach, spleen, kidneys, adrenal glands, intestine, mesenteric lymph node, urinary bladder, mammary gland, eye, skeletal muscle, bone marrow, skin with subcutaneous tissue, and spinal cord. Ovaries and testes, including the ductus deferens, were sampled and stored separately in formalin, to be analyzed in future projects on the reproduction of otters. Furthermore, the brains of the majority of the otters were saved separately in formalin for future investigations.

### 2.4. Mycology

DNA was extracted from three tissue slides (5 μm each) from formalin-fixed paraffin-embedded tissue. Fungal DNA was amplified using a broad range fungal qPCR targeting the ribosomal 28S rRNA gene using the primers 10f and 12r, as previously described by Rooms et al. [42]. Due to inhibition of the PCR reaction when the undiluted DNA elution was analyzed, as detected by an internal amplification control, the qPCR was also performed in a 1:10 dilution. While the PCR was negative from the undiluted specimen due to inhibition, the diluted samples amplified different fungal DNAs that could not be identified by Sanger sequencing of the PCR amplicons. All of the techniques used were described previously by Rooms et al. [42].

### 2.5. Bacteriology

Various organ samples, including opportunistically sampled swabs (swab set, sterile, Amies Medium, Covetrus DE GmbH, Hamburg, Germany) were taken for bacteriology (Supplementary Materials) and stored at −20 °C for the regular tissue and +4 °C for the swabs, until analysis. Cultivation was carried out at the Institute of Hygiene and Infectious Diseases of Animals, Justus Liebig University Giessen, Giessen, Germany, following the methods of Prenger Berninghoff et al. and Siebert et al. [43,44]. Grown colonies were subcultured and pure cultures were identified using standard morphological and biochemical methods for all samples, as well as MALDI-TOF mass spectrometry with the Microflex LT/SH instrument, as instructed by the manufacturer (Bruker Daltonics, Bremen, Germany) [45,46,47].

### 2.6. Virology

Lung tissue samples and swabs (Sigma Virocult, polyurethane tip 20 mL, Virocult fluid medium, Check Diagnostics GmbH, Westerau, Germany) were taken and stored at −70 °C until analysis. Furthermore, short sections of closed small intestinal loops were sampled and stored at −70 °C until analysis. Investigations were carried out at the Institute of Virology, University of Veterinary Medicine Hannover, Foundation, Hannover, Germany. A TaqMan real-time PCR was used for the detection of canine and feline parvovirus genomes [48]. Screening for morbillivirus-specific nucleic acid was performed by reverse transcription–polymerase chain reaction (RT-PCR) [49]. An established RT-qPCR protocol was applied for the detection of SARS-CoV-2 RNA [50].

### 2.7. Parasitology

The skin and fur of all otters were screened macroscopically for ectoparasites. Detected specimens were identified morphologically under a microscope (Olympus SZ61). The lungs, heart, liver, stomach, and intestines were routinely checked for endoparasites macroscopically during necropsy. All parasites found were first stored in water and were later transferred to 70% ethanol (Rotilab^®^ Container, PVC clear, Carl Roth GmbH, Karlsruhe, Germany). Small sections of closed intestinal loops and pieces of musculature (heart, tongue, diaphragm, and skeletal muscle) were stored at −20 °C until analysis. Endoparasites in the sections of the intestine were microscopically determined at the Institute for Parasitology, University of Veterinary Medicine Hannover, Foundation, Hannover, Germany. Additionally, samples of 10 animals harboring protozoal cysts in their muscles were representatively analyzed genetically using primers COC-1 and COC-2 [51], targeting the nuclear small subunit RNA of different coccidia, e.g., *Sarcocystis* spp. and *Toxoplasma gondii*, and the resulting amplicons were custom sequenced. Furthermore, the haemolytic serum samples of nine otters were available and screened as duplicates for anti-*Toxoplasma gondii* antibodies using the ID Screen^®^ Toxoplasmosis Indirect Multi-species Elisa (IDvet, Grabels, France). This test was considered adequate for the haemolytic serum samples, as it can be used for meat juice, besides plasma and serum. To enhance reliability, samples were screened with both the recommended dilution for serum (1:10) and meat juice (1:2), and were tested as duplicates in both dilutions instead of as single reactions. According to the manufacturer’s recommendations, samples with a S/P ratio ≤ 40% were classified as negative, with 40% < S/P < 50% as questionable, and with S/P ≥ 50% as positive.

### 2.8. Age Determination

Based on Heggberget [52], a lower or upper canine was taken to count the incremental cementum layers. The teeth were placed in a dissolution (disinfection dissolution, Caramba Chemie GmbH & Co. KG, Künzelsau, Germany) for 30–60 min during maceration, and the remaining surrounding tissue was removed. They were then stored in containers (Rotilab^®^ -Container, PVC clear, Carl Roth GmbH, Karlsruhe, Germany) with 70% alcohol for 24 h, and afterwards were rinsed in water for 24 h. The teeth were then transferred into separate cassettes (Universal-Embedding cassettes with a plastic lid, Engelbrecht GmbH, Edermünde, Germany) and fixed in 10% buffered formalin for another 10–24 h. After fixation, the teeth were rinsed in water again for 24 h. Decalcification was performed using a RDO^®^ Rapid Decalcifier (Apex Engineering Products Corporation, Aurora, IL, USA) for 24 h. Before cutting, they were rinsed in water for another 24 h. Such prepared teeth were cut into 16 μm thin slices using a microtome (SM2400 microtome, Leica) with a connected freezing stage at −11 °C (BFS-MP Series Freezing Stage, Physitemp Instruments Inc., Clifton, NJ, USA), and special glue was used to fix the teeth (MEDITE^®^ Cryo Embedding Medium, Medite Medical GmbH, Burgdorf, Germany). The slices were stained in 0.3% toluidine dilution for 15–20 s and then mounted on microscope slides (Thermo Scientific, Inc., Microscope Slides, Rockford, IL, USA) that had been previously coated with commercial gelatine. Coverslips (24 mm × 60 mm, 1#, Carl Roth GmbH, Karlsruhe, Germany) were attached after drying, using special glue (DPX Mountant for histology, Sigma-Aldrich Chemie GmbH, Taufkirchen, Germany). Examination was performed microscopically (Olympus SZ61) and age interpretation followed Sherrard-Smith et al. [53], presuming winter band formation and second dentition after 6 months of age.

## 3. Results

### 3.1. Location and Finding Data

**Figure 1:**
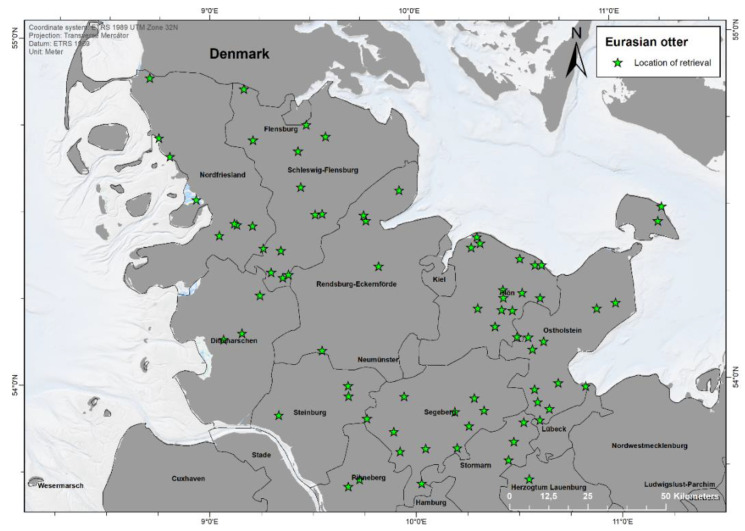
Finding locations of 85 otters found dead between 2015 and 2020 in Schleswig-Holstein, Germany. Map made with ArcGIS for Desktop 10.5 (© ESRI, Inc., Redlands, CA, USA).

A total of 92 otters were dissected from the following years: 2015 (*n* = 4), 2016 (*n* = 7), 2017 (*n* = 7), 2018 (*n* = 9), 2019 (*n* = 28), 2020 (*n* = 31), and unknown (*n* = 6). Finding locations were available for 85 animals, showing a clear concentration along the Eastern part of Schleswig-Holstein (Continental region) (Figure 1). In few cases, no location or year of finding of some otters provided by Kiel University was available, due to unknown reasons. Most findings occurred in the rural district Plön (*n* = 16), followed by Ostholstein (*n* = 15), Schleswig-Flensburg (*n* = 13), and Segeberg (*n* = 11). No otters originated from the urban municipalities Lübeck, Kiel, Neumünster, and Flensburg. Notably, in 2020, more sightings in the Western part of the federal state (Atlantic region) occurred compared to 2019.

Seasonal patterns were only compared for animals from 2019–2020, as they were collected strategically. Most otters were found from September to February, whereas fewer carcasses originated from March to August. Notably, no animals were found in April. The highest peaks in 2019 occurred in January (*n* = 4), September (*n* = 4), November (*n* = 5), and December (*n* = 4). In 2020, the findings peaked in January (*n* = 5), February (*n* = 5), September (*n* = 4), and October (*n* = 4).

### 3.2. Sex and Age

Regarding sexes, there was a general bias to males (*n* = 53) compared to females (*n* = 38); in one otter, the sex could not be determined. In 2019, there were equal numbers of males and females (both *n* = 14), whereas in 2020, almost twice as many males (*n* = 20) as females (*n* = 11) were found. The teeth from 65 otters could be processed for cementum aging (Table 1). In the remaining cases, the teeth were either missing or irreversibly damaged. Two individuals were cubs. The pulp cavity of nine otters was wide open, indicating juveniles in their first year [54]. More than 60% of the otters were younger than three years old. Moreover, more than 80% of the otters were younger than three and a half years old. The oldest animal was estimated to be a maximum of seven and a half years old.

### 3.3. Pathological Findings

#### 3.3.1. Nutritional Status

**Figure 2:**
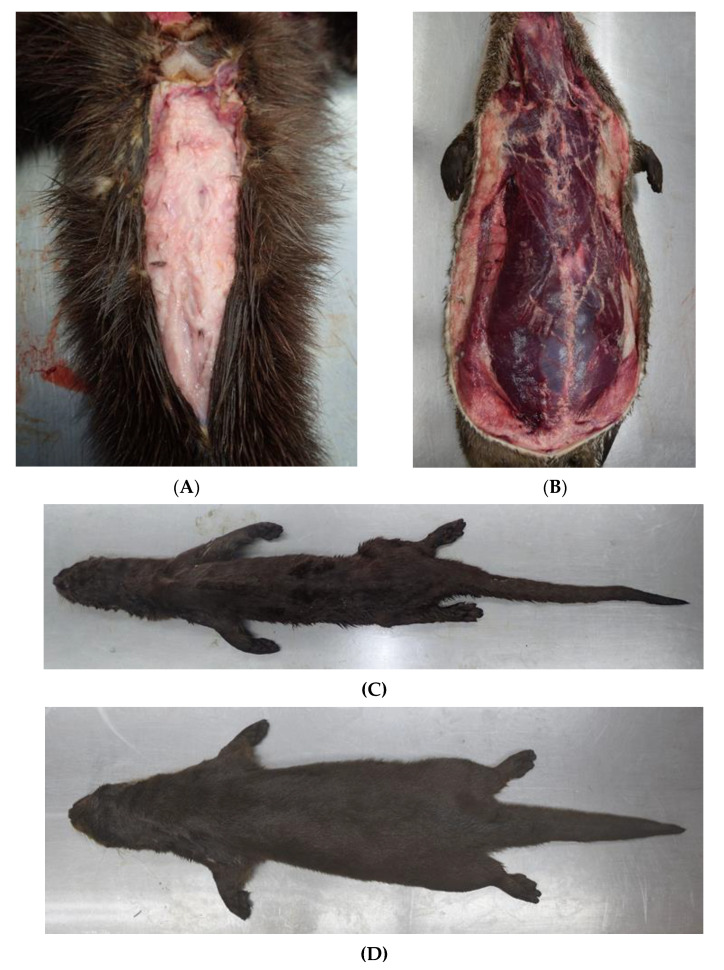
Otter in good nutritional status with a lot of subcutaneous fat in the tail radix region (**A**) and along the body (**B**). Severely emaciated otter (**C**) in comparison to an individual in good body condition (**D**).

The majority of otters collected before 2019 were deskinned for taxidermy and body parts were missing at the time of dissection. This also applied to 11 animals in 2019 and 12 in 2020. In cases where the body condition could be assessed, most otters were in good nutritional status (*n* = 52), and some were of a moderate (*n* = 14) and poor nutritional status (*n* = 7; Table 2 and Figure 2).

An overview of all of the histopathological findings is given in Table 3. Quantity equals all findings made, which therefore sometimes resulted in several diagnoses (e.g., pneumonia) for certain organs in individual otters.

#### 3.3.2. Respiratory Tract

**Figure 3:**
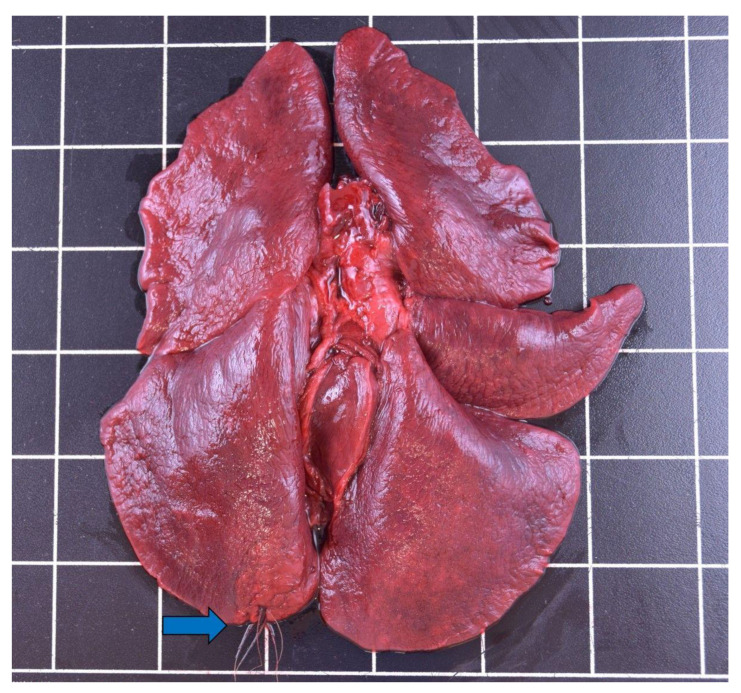
Plant fibers (blue arrow) perforating the lung of a male otter from Schleswig-Holstein.

Lymphohistiocytic and plasmacytic, interstitial, and eosinophilic pneumonia were detected most often (*n* = 12), and affected predominantly females (*n* = 4) between 1.3–5.5 years and one male (2.5–3.5 years). This was followed by granulomatous pneumonia (*n* = 7), twice with intralesional foreign material (Figure 3), which disproportionately affected male otters (*n* = 6) between 0.75–3.5 years compared to females (*n* = 2), for up to 5.5 years.

**Figure 4:**
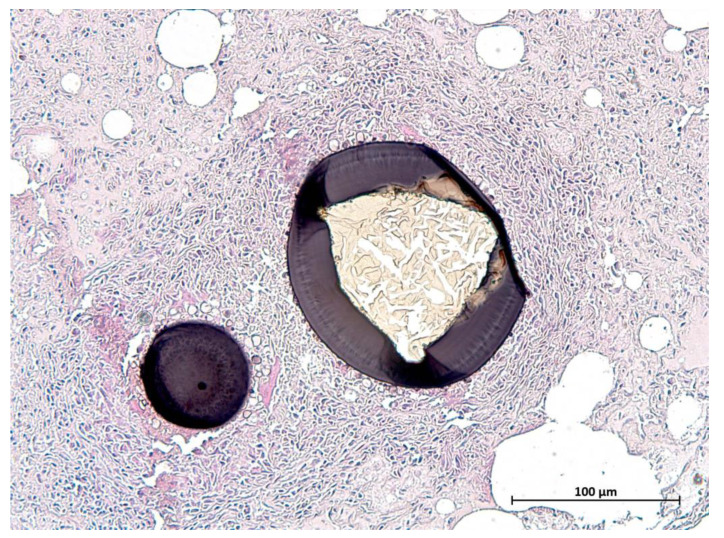
Fungal adiaspore in the lung tissue of an otter from Schleswig-Holstein.

Bronchopneumonia was seen in two males of an unknown age, in one case together with intralesional fungal spores (Figure 4). An additional female (2.5–4.5 years) likewise showed bronchopneumonia with intralesional structures suggesting adiaspores [55]. Using a broadrange PCR assay fungal DNA of the typical agent of Adiaspiromycosis, *Adiaspiromyces* could not be amplified, probably due to inhibition of the PCR, a frequent problem in formalin-fixed and paraffin-embedded tissue, and the presence of other fungal DNA from different potentially contaminating fungi.

Pyogranulomatous pneumonia was diagnosed in one male otter (2.5–3.5 years). Nonpurulent pleuritis occurred in one male (2.5–3.5 years). Another male otter had a foreign body (plant fiber) in its lung and associated severe growth of bacteria on the pleura pulmonalis, and septic spread of bacteria was assumed. A female otter (0.6–1.7 years) showed non suppurative bronchiolitis. Hemorrhages only occurred in animals that died as roadkill, and were therefore attributed to blunt trauma.

#### 3.3.3. Cardiovascular System

Fibrosis of the myocardium, together with severe loss of cells and focal metaplastic bone development were seen in one male otter (3.5–5.5 years). Protozoal cysts were found in one female (1.3–2.25 years) and one male (1–2 years) otter.

#### 3.3.4. Thoracic Cavity

A male otter (2.5–3.5 years) displayed nonpurulent serositis (diaphragm) and was also affected by pneumonia (nonpurulent and granulomatous) and pleuritis. In a third male (1.5–3.5 years), an interstitial edema was found in the diaphragm. Protozoal cysts in the diaphragm were present in four female (~0.5–3.5 years) and four male (~0.5–5.5 years) otters (see also 3.6 Parasitology).

#### 3.3.5. Alimentary Tract

**Figure 5:**
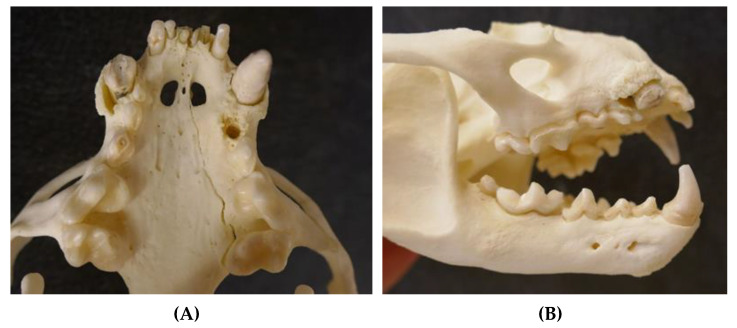
Severely worn-down teeth of an adult male otter (5.5–7.5 years) from Schleswig-Holstein; some teeth are even missing ((**A**) ventral view upper jaw and (**B**) lateral view skull).

**Figure 6:**
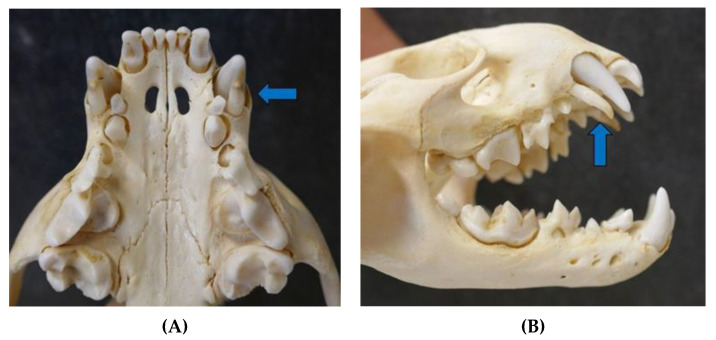
Erupted permanent teeth and deciduous teeth (blue arrow) in a male otter cub from Schleswig-Holstein ((**A**) ventral view upper jaw and (**B**) lateral view skull).

The teeth were severely worn and showed dental calculus in the oldest male (Figure 5) of the study (5.5–7.5 years). In general, some teeth of individual otters were fractured or missing, which was attributed to blunt trauma after vehicle collision. Deciduous and permanent teeth erupted in one male cub (Figure 6).

Protozoal cysts in the lingual musculature were found in six female (~0.5–5.5 years) and five male (~0.5–3.5 years) otters (see also 3.6 Parasitology). A male of unknown age showed both erosions and bleedings of the gastric mucosa, as well as ulcerative gastritis histologically. This was a reported roadkill. An adult female (2.4–4.5 years) with prominent tits and placental scars displayed lymphohistiocytic gastritis. Another female of unknown age that had reportedly died during veterinary care after vehicle collision had erosions of the gastric mucosa. A male cub that had died during rehabilitation after being found abandoned and emaciated had a submucosal gastric edema. The same animal suffered from acute purulent and necrotizing enteritis. Portal fibrosis occurred in the liver of one male (unknown age) and one female (2.5–4.5 years). The animals also suffered from granulomatous pneumonia and bronchopneumonia. The male otter displaying ulcerative gastritis also had cholelithiasis, associated with fibrosis, in its liver. Portal hepatitis was diagnosed in a female otter (0.5–2.5 years).

#### 3.3.6. Urinary and Reproductive Tract

**Figure 7:**
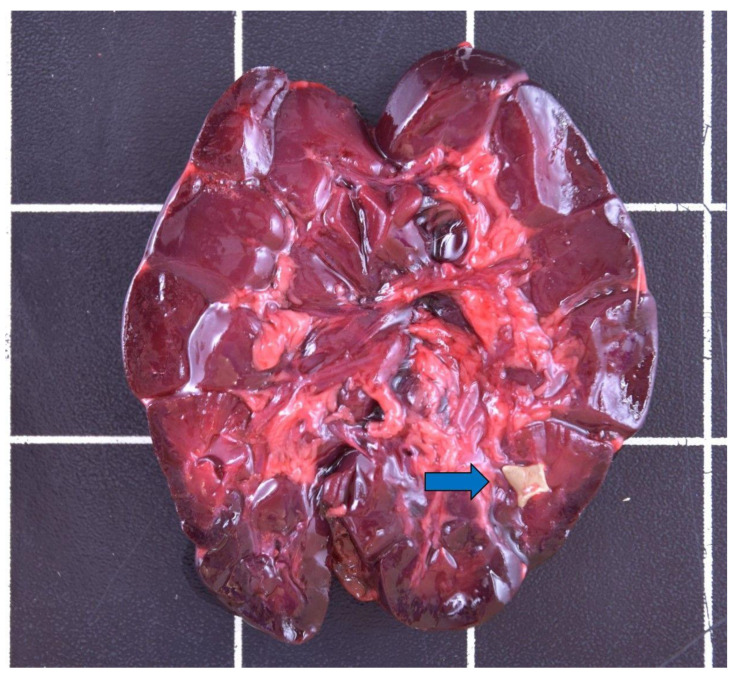
Nephrolithiasis (blue arrow) in the kidney of a male otter from Schleswig-Holstein.

Nephrolithiasis and urolithiasis (Figure 7) were found in four males (1.9–7.5 years) and one adult female (2.5–3.5 years) with placental scars.

**Figure 8:**
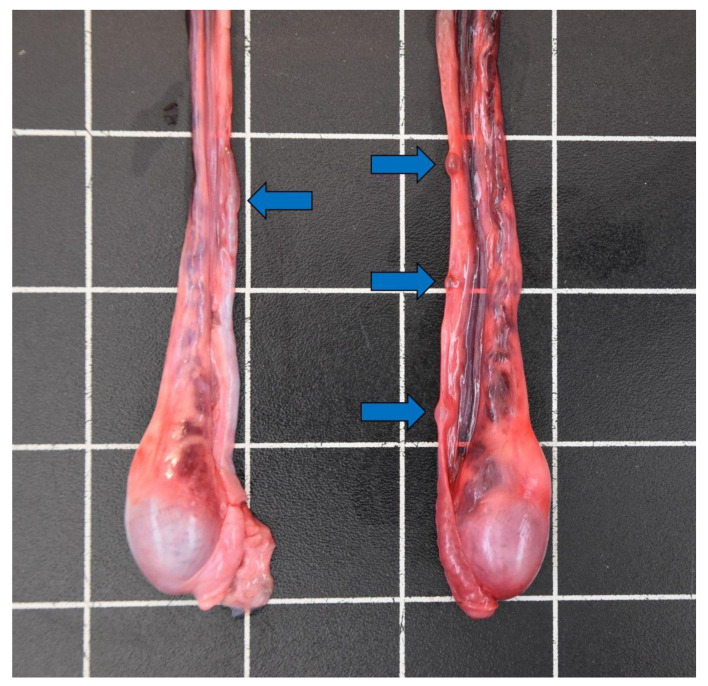
Cysts on the vas deferens (blue arrows) of a male otter (5.5–7.5 years) from Schleswig-Holstein.

Cysts on the vas deferens (Figure 8) were present in 18 male otters (~0.5–7.5 years). Most findings of cysts occurred in 2020, which most likely represents the lack of awareness of such alterations during necropsies in earlier years.

Signs of reproduction in females were prominent tits (*n* = 9), placental scars (*n* = 4), and pregnancy (*n* = 2). Two individuals only showed placental scars (1.3–2.25 and 3.5–5.5 years) and five only had prominent tits (1.5–5.5 years). The pregnant females also had prominent tits (2.2–3.3 years).

#### 3.3.7. Skin and Subcutis

An emaciated and dehydrated male (3.25–4.25 years) with behavioral disorder reportedly could not use its hind legs anymore, which led to alopecic patches and abrasions of the inguinal skin and on the hind legs, as the animal simply dragged them along. Dermal lesions could further be detected on the paws and the prepuce. The otter had been captured alive and died later on. Bleeding of the spinal cord was also detected histologically in this individual. Dermal fibrosis was found in a female (1.5–2.5 years), probably indicating a healed lesion. Subcutaneous bleeding in another female most likely resulted from blunt trauma after vehicle collision. Paired punctual lesions 1–2 mm in diameter and 15–25 mm apart were found on the foot pads (*n* = 5) and in the anogenital region (*n* = 1) of six otters, and were interpreted as bite marks. The affected animals included three males (~0.5–2.5 years) and three females (2.3–4.5 years). Three of those otters also had pneumonia and one had gastritis.

#### 3.3.8. Musculoskeletal System

The vast majority of otters died as roadkill and displayed simple to comminuted fractures, especially of the cranium, but also of the spinal column, ribs, pelvis, and various long bones. Severe lacerations of the surrounding skeletal muscles were typically associated with bone fractures. Notably, most road-killed animals seemed to have been hit in the head region by vehicles. A few otters were severely emaciated, showing atrophy of the skeletal muscles. These included one orphaned male cub, at least three male juveniles (~0.5–2 years), the adult male (3.25–4.25 years) with behavioral disorder, two males of unknown age, and a juvenile female (~0.5 + years). Protozoal cysts without adjacent reactive changes in the skeletal muscles of different parts of the body were present 14 times (see also 3.6 Parasitology). These cases included eight female (0.5–5.5 years) and six male (~0.5–4.25 years) otters. One male otter (1–2 years) had periostal hemorrhages, which were attributed to blunt trauma after vehicle collision. One pregnant female otter (2.3–3.3 years) had an additional sixth phalanx on the left foreleg.

#### 3.3.9. Central Nervous System, Eyes, and Ears

In most otters, the skull, including the brain, was severely destroyed due to blunt trauma, limiting morphological assessment. Hemorrhages of the spinal cord were seen in three animals, including one female (0.5–2.5 years) and one male (1–2 years) that had died as roadkill. The third individual was the adult male with behavioral disorder, whose spinal nerves did not show any histological alterations. Synechiae with mild endophthalmitis and associated atrophy of the retina, probably indicating blindness of this eye, were diagnosed histologically in one adult female (4.2–5.2 years) that had died as roadkill. Hard ticks infested the ears and surrounding area in seven otters. These cases included four males (0–4.25 years) and three females (1.5–5.5 years).

#### 3.3.10. Hematopoietic and Endocrine System

Follicular hyperplasia was found in the lymph nodes (*n* = 6), tonsils (*n* = 3), and spleen (*n* = 1). Follicular hyalinosis with concurrent follicular depletion occurred in lymph nodes (*n* = 21), spleen (*n* = 20), and Peyers patches (*n* = 1). This included 20 males (0–7.5 years) and 16 females (0.5–5.2 years). Hemosiderosis of the lymph nodes was found five times, including in the adult male with behavioral disorder, whereas all other animals were roadkill. In one emaciated cub with gastric edema and enteritis, the lymph nodes showed lymphatic depletion, possibly indicating immuno suppression.

Thyroid follicular cysts were seen in four male otters (1.9–5.5 years) and one female individual (1.5–2.5 years). In 10 cases, lymphohistiocytic adrenalitis was present, and was apparent in seven male (1.3–3.5 years) and three female otters (2.2–4.5 years).

### 3.4. Microbiology

Various tissue samples of 47 otters were screened, detecting 37 different species or genus of bacteria and fungi (Table 4). Only moderate to severe levels of growth were taken into consideration. Among the species/genera mostly detected were *Pseudomonas* spp. and α- and γ-hemolytic streptococci. Potential zoonotic agents included *Clostridium perfringens* and *Vagococcus lutrae*.

### 3.5. Virology

Pan-Morbilli RT-PCR for distemper virus infection was negative for all 41 out of the 92 tested otters. PCR for Canine and Feline Parvovirus infection was conducted for 38 animals; all of which tested negative. In 19 otters from 2020, no SARS-CoV-2 infection could be detected via RT-PCR.

### 3.6. Parasitology

**Figure 9:**
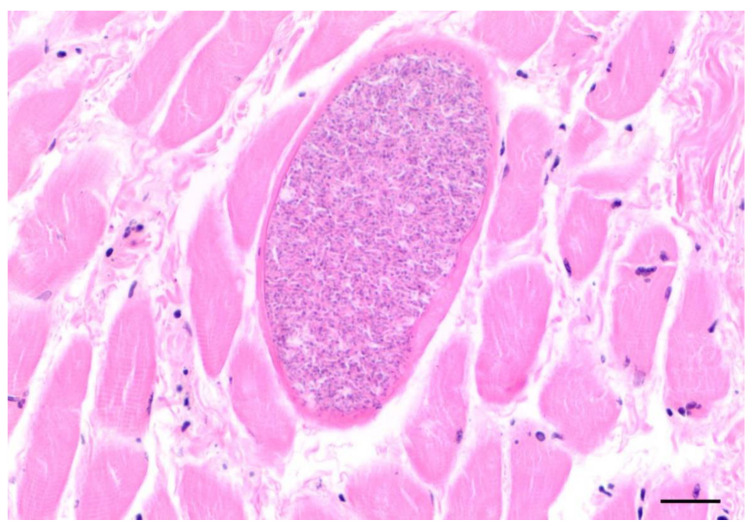
Cyst of *Sarcocystis lutrae* in the tongue of an otter from Schleswig-Holstein (scale bar 100 µm).

Protozoal cysts in the skeletal muscles, myocardium, lingual musculature, and diaphragm were seen in 25 otters histologically (Figure 9). Females (*n* = 13) of all ages were affected (0.6–5.5 years), which was also the case for males (*n* = 12; 0.5–4.25 years). Samples of 10 animals, which were representatively analyzed genetically, revealed *Sarcocystis lutrae* infection in all of the tested otters. Due to similar cyst morphology in the size and cyst wall appearance, the same pathogen was assumed in the remaining histologically observed cases.

Serological testing of available haemolytic sera for anti-*Toxoplasma gondii* antibodies yielded positive results in both duplicate setups (each one for serum and meat juice) in seven out of nine otters, while the two other individuals were negative in all reactions. The seropositive otters included three females (0.5–4.5 years) and four males (0.5–7.5 years). In contrast, no *T. gondii* cysts were detected in any tissue samples histologically. In the sections of intestinal loops, quite decomposed endoparasites were found in three otters. In one female (1.6–2.6 years), a nematode resembling *Oswaldocruzia filiformis* was observed. Three trematode specimens resembling *Isthmiophora melis* were found in another female (0.5–2.5 years). Additionally, two specimens resembling *Corynosoma* spp. were detected in a male otter (1.9–2.9 years). Ticks occurred in seven otters, ranging from a few individuals up to 13 ticks infesting one animal. This included three females (1.5–5.5 years) and four males (0–4.25 years). The highest tick burdens were 13 parasites each on a juvenile male and a cub. Besides mostly larval and nymph stages, few adult ticks were present. Morphologically, all of the ticks were identified as hard ticks of the genus *Ixodes*.

### 3.7. Causes of Death

The most common cause of death among the study animals was blunt trauma after vehicle collision (roadkill) (Table 5). The loss of the mother led to starvation in at least two juvenile otters, and emaciation and starving contributed to the death of an adult male otter with behavioral disorder. One female with a foreign body in the lung probably died due to septic spread from bacteria. In some cases, the cause of death could not be determined. This included animals that had been processed by taxidermists in the past, where only incomplete carcasses could be observed. One animal from 2020 was already skeletonized when found, making it impossible to investigate it any further.

## 4. Discussion

The number of otters found dead in Schleswig-Holstein has been rising continuously over the last decades, indicating a growing population [56]. The disparity in the number of reported carcasses and those received for dissection has decreased due to improvement in the network strategy. The finding locations showed a spatial aggregation along the Eastern part of Schleswig-Holstein (Continental region), which coincides with the known distribution routes of the local population [4,6]. It is further possible that habitat structures, such as large waterbodies in the east, are more attractive for otters, compared to mainly agricultural pasture in the west. Temporal trends indicated that most otters were found during the fall and winter months, whereas this differed among the years. Prolonged night-time seems to correlate with more otters getting killed on roads, which could be due to rising activity levels of otters, but also meteorological patterns [14,15,57,58,59,60]. There was an overall male bias among the study animals, whereas this differed among the years. Such bias is known from the Eastern German population [17,32,37,61], but is also internationally common [14,15,16,19,58,59,62,63]. Possible overrepresentation of male otters, e.g., due to larger territories and higher energetic demands, needs to be considered [17,59,64]. Despite no strict seasonal reproduction and discontinuous banding patterns, counting incremental layers is the most reliable source for aging otters nowadays [52,53,54,65]. The average age of most otters in this study was between 1–3 years, indicating many subadult–adult animals [39,66]. Subadult otters, especially males, looking for new territories, show greater migration behavior compared to adult individuals, which might explain the higher prevalence of this age group among the study animals [17]. This coincides with mean ages in for example Scottish or Spanish populations [67,68], but contradicts the Eastern German one, where most otters were older than three years [17,32,37,61]. Other international studies used less specific age determination methods, which limits comparability herein [14,15,19,59,62,69].

The vast majority of otters displayed good body condition, which was also seen in the Eastern German population [32,61]. There is a known bias towards otters with good nutritional status that are killed on roads, which needs to be taken into consideration [14,62,67,68,70]. Some individuals were emaciated, which might be attributed to the loss of their mother prior to death in the cases of juveniles [15]. Another emaciated otter was an adult male that showed behavioral disorder and could not move its hind legs anymore, which probably led to starving due to inability to catch prey. Most carcasses were in a moderate state of decomposition, still allowing for extended processing and sampling. Nonetheless, the trauma-induced fractures, ruptures, and lesions of organs limited the interpretation during necropsy [15]. Freezing of carcasses and tissue samples prior to microbiological investigation should preserve bacterial and fungal cells, but cell damage is unavoidable [71]. Successful culturing depends on the genus or species, and might influence microbiological results concerning amount and diversity.

The lungs of the study animals were predominantly affected by various types of pneumonia; nonpurulent forms (*n* = 12) were detected most often. Infectious and non-infectious causes have to be considered [72]. No parasites were observed macroscopically or histologically in the lungs. Furthermore, RT-PCR was negative in all samples screened for distemper virus and SARS-CoV-2. Still, Eurasian otters have been infected with distemper virus in Denmark and Germany in the past [19]. Other otter and mustelid species seem to be susceptible to Canine distemper virus (CDV) and may even function as a vector [19,73,74,75,76]. Along shorelines, otters share their habitat with marine mammals, risking the spillover of CDV to, for example, seals [77]. Harbour seals (*Phoca vitulina*) in Germany have suffered from epizootics of phocine distemper virus (PDV) in the past [78,79]. In Italy, several Eurasian otters displayed clinical infections with a dolphin morbillivirus [18]. It is therefore of great importance to survey potential morbillivirus outbreaks in the future, also due to potential interspecies spill-over. Despite no coronavirus infection being found among the study animals, and no infections for *Lutra lutra* being documented at the time of writing, the first infections in captive Asian small-clawed otters (*Aonyx cinereus*) were already reported ([80], assessed 18 August 2021). As it is known, other mustelids function as an important vector for the zoonotic pathogen, displaying mild to severe symptoms [21,22,81]. More research needs to be done to investigate the potential role of otters in the spread of zoonotic viruses like SARS-CoV-2. Purulent (*n* = 1) and granulomatous pneumonia (*n* = 7) occurred together with intralesional foreign material in at least three cases. The foreign material could be determined as plant fibers and insect remnants in two otters. In one of those otters, the plant fibers were granulating out of the caudal tip of the left lung, which probably caused infection of the thoracic cavity. This indicates that aspiration of such foreign bodies can lead to lung infection and even fatal septic spread of bacteria. Likewise, septic spread of bacteria might lead to pneumonia in otters after inter- or intraspecific fights in otters [15,76,82,83]. Three of six otters with bite wounds in this study also had pneumonia (nonpurulent or granulomatous), in one case involving intralesional foreign material in the lungs. Bronchopneumonia (*n* = 3) was associated with intralesional fungal adiaspores in two animals and was interpreted as adiaspiromycosis. Caused by fungus *Adiaspiromyces*, previously known as *Emmonsia* spp. [84], it is known to affect the lungs of various members of the Mustelidae, including otters [26,85,86,87], even with a fatal outcome [25]. Both affected animals from the study were reported roadkills; the nutritional status could not be assessed in one animal, but was good in the other individual. It was therefore assumed that the fungal infections had not severely impacted the health of the two otters. The main bacteria cultured from purulent and granulomatous pneumonia, as well as bronchopneumonia were *Pseudomonas* spp. and α- and γ-hemolytic streptococci. The mentioned streptococci, together with findings such as *Pantoea* spp. and carnobacteria, most likely represented commensal bacteria. As a pathogen known to severely affect other mustelids [26,88], *Pseudomonas aeruginosa* was already isolated from Eurasian otters in Israel [89]. Furthermore, unspecified pseudomonades were found in the feces of five Eurasian otters from Portugal [23]. Dermal lesions in a *Lontra canadensis* specimen were associated with *Pseudomonas putrefaciens* infection [76]. Unspecified streptococci were isolated from a Danish otter [19]; *Streptococcus porcinus* was found in the feces of an otter from Portugal [23]. Two study individuals contained *Streptococcus canis* in the lungs and *Streptococcus dysgalactiae* in the associated pulmonal lymph node, respectively. Simpson [15] made the same finding in otters with and without associated macroscopical lesions in the lungs. Pneumonia as a clinical disease may potentially lead to the death of Eurasian otters [19], whereas this was not the case in any study animal from Schleswig-Holstein. Pleuropneumonia occurred in one otter, whereas severe growth of bacteria on the pleura was detected in one individual that had a plant fiber perforating the lung, indicating a causative origin of infection. In both cases, no specific bacteria were isolated. Death due to septic spread of bacteria was assumed in another case of diaphragmatic serositis, whereas no specific bacteria were cultured. Simpson [15,83] described similar infections of the pleura due to septic spread of bacteria after intra- and inter-specific interactions of otters and other mustelids, such as American mink (*Neovison vison*). None of the affected study animals had visible bite wounds.

The etiology of myocardial fibrosis with associated cell loss (*n* = 1) could not be determined. The detection of anti-*T. gondii* antibodies in seven study animals coincided with previous findings from Great Britain [35] and Spain [36]. Smallbone et al. [90] suggested seroprevalence in Eurasian otters was influenced by certain abiotic and biotic factors, and the highest infection occurred on arable land. Agricultural run-off into aquatic ecosystems, its semi aquatic lifestyle, and opportunistic feeding behavior probably expose *Lutra lutra* to infection [26,35,90]. Despite mustelids being considered as an accidental host for *T. gondii* [26], several cases of fatal toxoplasmosis were detected in captive Asian small-clawed otters [91], raising the question whether infected wild otters displaying clinical disease might be overlooked in studies mainly targeting roadkilled individuals [35]. Recent findings in North American river otters indicate the brain as an additional valuable source of detecting infections with *Toxoplasma* [92], together with lymphatic tissue, among other, as seen in Asian short-clawed otters [91].

Gastric erosions and ulcerations of varying degrees, sometimes associated with bleeding, reportedly occurred in Mustelidae [26], as was also seen among the study animals (*n* = 3). In otters, stress-induced factors can lead to such findings [15]. Veterinary care of orphaned or injured otters in the study might have also contributed to the development of gastric erosions and edema (*n* = 2). Ulcerations, erosions, and bleeding of gastric mucosa, together with emaciation, indicated starvation related pathology in one otter. Similar to the case of one otter with bacterial infection in Great Britain [15], nephro- and cholelithiasis were also diagnosed in the latter animal. Gastritis (*n* = 1) could not be related to any systemic infection or disease, whereas signs of recent pregnancy might have been an indication of immune suppression in the affected adult female. Purulent and necrotizing enteritis was seen histologically in one otter, whereas no bacterial infection was assessed. Other bacteria that could not be cultured, as well as infectious and non-infectious agents, need to be considered. *C. perfringens* was cultured from the intestines of three study animals, whereas no related pathology was observed. In Portugal, approximately 10% of the studied otter fecal samples contained *C. perfringens*, whereas no additional conclusions on potential related pathologies could be drawn [23]. *C. perfringens* related enterotoxemia was described in captured North American River otters during the process of translocations, probably induced as the result of stress and bacterial overgrowth [76] or through dysbacteria and enterotoxemia in other mustelids [93]. Small intestine samples from 38 otters tested negative for parvovirus infection, which again agrees with the results from Eastern Germany [32]. North American River otters seemed to carry a variety of parvovirus [75]. This was also the case for Eurasian otters in Italy, which even displayed coinfections with different parvoviruses [20]. Three otters in the study group had endoparasites in the examined section of their intestines, whereas advanced decomposition only allowed for suspected identification. One specimen of suspected *O. filiformis* in an otter was probably derived from amphibian prey [94]. *I. melis* is a trematode with an obligate intermediate host life cycle and zoonotic potential [32,95]. Infections in otters and other mustelids were reported in Germany, Austria, and countries in the east [31,32,96,97]. Two specimens were identified as suspected *Corynosoma* spp., of which several species are known to infect *Lutra lutra* [33,34,98]. Still, some species might also be transient parasites infesting prey species of otters [76,94,98]. *Corynosoma strumosum* infected Eurasian otters in Ireland, and, on the other hand, is commonly found in German Harbour Seals [34,41].

Hepatic fibrosis indicated a chronic alteration process of various origins in two otters [72]. Hepatitis occurred once, and the etiology thereof could not be finally determined. A correlated infection most likely led to the development of cholelithiasis and nephrolithiasis in an otter with gastric alterations [72]. Renal and uric calculi occurred in a total of five otters in the study. Both males (*n* = 4) and females (*n* = 1) were affected. Nephrolithiasis is a common finding in Eurasian otters both in the wild and in captive individuals [14,15,19,26,62,99,100]. Age-relation is at least suspected in some cases [14,100]; the age of the study animals ranged from 1.9–7.5 years. As otters mainly represent a piscivorous feeding type and also excrete a lot of uric acid, their purine metabolism might partly explain the etiology of renal calculi [26,99,100]. Cysts of different size on the vas deferens were detected in 18 male otters of all ages. Roos et al. [101] assumed a contaminant-related pathology, which could not be finally determined in this study. Four females displayed one to three placental scars, indicating former pregnancy [57,102], whereas two were pregnant, carrying one and two fetuses, respectively. One female carrying two fetuses had an additional mass in one uterus horn, only containing brownish fluid, which was interpreted as a fetus in resorption. Female otters reach sexual maturity with approximately 1.5 years of life [39], whereas mostly older individuals reproduced in the Eastern German population [37,66]. Reproduction rates are generally estimated as low in *Lutra lutra*, and pre- and postnatal mortality seem to be high [14,37,57,66,69,70,102,103], as also seen by the fetus in resorption in one of the study animals. The mean litter size differs among populations and was, for example, about 1.7 cubs per female in Denmark [102] compared to 2.36–2.7 cubs per female in Eastern Germany [32,37,66].

An isolate of *V. lutrae* was obtained from the intestine and associated mesenteric lymph node from an emaciated otter with dermal lesions and abrasions. First described in a Eurasian otter [24], this pathogen is known to have a zoonotic potential [104,105]. Bite wounds were detected in six otters in total, which could be seen as a sign of a growing population, but also interaction with other species [14,62]. Protozoan cysts in various sites of lingual musculature, skeletal muscle, heart, and diaphragm were found in 25 otters in the present study, of which the samples of 10 otters could genetically be identified as *S. lutrae*. The parasite was detected in Eurasian otters and other mustelids in Nordic and Eastern European countries [29,30,106,107]. Typically infesting intermediate hosts as cysts in muscled structures, top predators such as *Lutra lutra* probably resemble accidental hosts [29,106]. Polydactilia (*n* = 1) most likely represented an incidental finding among the study animals. Hemorrhages in the spinal cord were seen in one individual, whereas the spinal nerves did not show any alterations. Even without signs of blunt trauma, it cannot be ruled out that this otter had been hit by a vehicle in the past, which also might have led to the observed behavioral disorders. Madsen et al. [19] mention a blind otter in their study, as was probably the case with one animal from Schleswig-Holstein, whereas this most likely did not have any serious issues for the individual. So far, mainly hard ticks of the species *Ixodes hexagonus* have infested Eurasian otters in Europe [27,28,58], whereas *Ixodes ricinus*, *Ixodes trianguliceps*, and *Ixodes canisuga* have also been found [14,27,32]. Among the study animals, only seven otters had ticks, identified as the genus *Ixodes*, ranging from one to 13 parasites per individual, compared to 77 parasites in, for example, East Germany [27]. They were almost exclusively found in the head region, close to the ears [27]. According to Sherrard-Smith et al. [28], malnutrition in otters sometimes correlates with higher tick burdens. Two of the study otters with ticks were emaciated, including a cub with 13 parasites as the highest burden. Potential bias in ectoparasites on otters might be an overrepresentation of healthy animals and the fact that such parasites leave their host shortly after death [27,28,32]. Hyperplasia of lymphatic organs such as lymph nodes, spleen, or tonsils were seen in many otters in the study, most likely representing an acute immunostimulating process such as that associated with the various detected pneumonia [72]. Follicular hyalinosis typically is found in chronically immunostimulation and is usually associated with a loss of follicular lymphatic cells [72]. Accordingly, many of the study animals had dealt with infections at some point in their lifetime, whereas retrospective assessment of their severity and impact on the health is not possible. Overall, lymphoid depletion might have been the result of immune suppression due to emaciation (*n* = 1). Adrenalitis can develop after bacterial or viral infection, whereas hyperplasia of the adrenal glands is most likely age related [72]. It remains unclear whether the detected alterations of the adrenal glands in the study animals posed a serious threat to the health of the otters, or whether they represented incidental findings of little clinical relevance. In summary, the majority of detected findings/changes did not necessarily have a measurable impact on the health of the examined otters, with few exceptions. Additional challenges were the limited assessment of endoparasites due to colliding interests in diagnostics, or the lack of suitable blood samples to perform serology testing to screen for former viral infections.

The main cause of death in most otters was roadkill, which reflects various national and international studies [14,15,16,17,19,26,32,37,58,59,62,63,108,109]. Furthermore, species protection plans and risk assessments list traffic-related mortalities as major threats for Eurasian otters [2,3,61,110,111,112]. Important reasons for otters leaving the riverbed to cross roads are improperly constructed bridges, including missing passageways underneath [2,61,113]. It is generally agreed that healthy otters in good nutritional status are overrepresented in studies using roadkill, as sick animals most likely hide themselves and the carcasses are less likely to be found [14,17,59,62,67,70]. Furthermore, the number of animals found on roads probably only represents a percentage of the total population that actually died [32,114,115]. Starving might be an overlooked natural cause of death with great significance in Eurasian otters [67,70,116]. It has been shown that when working with wildlife, to investigate all animals found dead is the best strategy to assess the health status of a population [41], and this is certainly also the case for the protected and elusive Eurasian otter [13]. If conducted over a longer period of time, standardized necropsies can continuously feed data- and bio-banks, which will allow future retrospective assessments in a spatiotemporal frame. Besides biometric data, information on age and sex, or the detection of the cause of death, the authors therefore strongly propose extended sampling protocols (Figure S1: Dissection Protocol-Otter). As top predators in freshwater ecosystems, Eurasian otters, for example, serve as excellent indicator species for contaminant studies [117]. In addition, a dietary analysis of the gastrointestinal content of otters found dead may be extremely helpful to support solving the human−otter interface (fisheries) by setting baseline data of what specimens in certain regions of interest might feed upon [118].

## 5. Conclusions

Monitoring the Eurasian otter via necropsies of dead specimens is a valuable tool to assess various parameters of population health. Still, shortcomings in performing diagnostics and certain bias limit the value of results and the subsequent interpretation. In Schleswig-Holstein, the otter population still seems to be growing, mainly consisting of young adult, but immature, individuals to date. Overall, most examined otters were in a status of good health, whereas this might be an overrepresentation. The main threats for otters in Northern Germany are collisions with vehicles, addressing the general need for safe passage for wildlife on roads. Natural causes of death might be underrepresented. It is of great importance to continue the monitoring, which will allow for insights into long-term population trends that could benefit the management of this protected and charismatic species.

## Figures and Tables

**Table 1:** Results of cementum aging of 65 otters found dead in Schleswig-Holstein.

Age of Otters [Years]	Number of Otters								
	Unknown Years			2015–2018		2019		2020	
	Male	Female	ND ^1^	Male	Female	Male	Female	Male	Female
<0.5	/	/	/	/	/	1	/	1	/
0.5–0.75	/	/	/	2	1	1	1	3	1
0.5–2.5	/	/	1	1	1	2	2	1	1
1.25–2.9	/	/	/	2	3	5	1	5	1
1.5–3.5	/	/	/	/	/	/	1	2	1
2.2–3.5	/	/	/	1	/	1	2	3	3
2.4–4.5	/	1	/	/	3	/	1	/	/
3.5–5.5	1	/	/	/	1	/	2	1	3
5.5–7.5	/	/	/	/	/	/	/	1	/

^1.^ ND = not determined.

**Table 2:** Nutritional status of otters found dead in Schleswig-Holstein.

Nutritional Status	Number of Otters				
	Unknown Years	2015–2018	2019	2020	Total
good	5	8	17	22	52
moderate	1	7	3	3	14
poor	/	3	2	2	7
ND ^1^	3	6	6	4	19

^1^ ND = not determined.

**Table 3:** Histopathological findings in Eurasian otters from Schleswig-Holstein between 2015–2020.

	Quantity				
Morphological Findings	Unknown Years	2015–2018	2019	2020	Total
Respiratory tract					
Pneumonia (purulent)	/	/	/	1	**1**
with intralesional foreign material	/	/	1	1	**2**
Pneumonia (nonpurulent)	/	5	5	2	**12**
Pneumonia (granulomatous)	1	2	3	1	**7**
with intralesional foreign material	1	/	1	/	**2**
Bronchopneumonia (nonpurulent)	1	/	2	/	**3**
with intralesinonal fungal spores	1	/	1	/	**2**
Bronchiolitis (nonpurulent)	/	/	1	/	**1**
Pleuritis (nonpurulent)	/	/	1	/	**1**
with bacteria pleura pulmonalis	/	/	1	/	**1**
Parenchymatous hemorrhages	/	/	3	3	**6**
Cardiovascular system					
Myocardial fibrosis with cell loss	1	/	/	/	**1**
Protozoal cysts myocard	/	1	1	/	**2**
Thoracic cavity					
Diaphragmatic serositis	/	/	1	/	**1**
Diaphragmatic edema	/	/	/	1	**1**
Protozoal cysts diaphragm	/	3	3	2	**8**
Alimentary system					
Gastritis	/	/	1	/	**1**
Gastritis (ulcerative)	/	1	/	/	**1**
Erosions of gastric mucosa	/	1	1	/	**2**
Gastric edema (submucosal)	/	/	/	1	**1**
Enteritis (purulent)	/	/	/	1	**1**
Portal hepatitis	/	/	1	/	**1**
Portal fibrosis	2	/	/	/	**2**
Cholelithiasis	/	1	/	/	**1**
Protozoal cysts lingual musculature	/	1	8	5	**14**
Urinary and reproductive tract					
Nephrolithiasis	/	2	1	1	**4**
Urolithiasis	/	/	/	1	**1**
Cysts vas deferens	/	2	4	12	**18**
Placental scars	/	1	2	1	**4**
Skin and subcutis					
Epidermal hyperplasia	/	/	/	1	**1**
Dermal fibrosis	/	/	1	/	**1**
Subcutaneous bleeding	/	/	1	/	**1**
Bite wounds	/	2	2	2	**6**
Musculoskeletal system					
Hemorrhages periostal	/	/	/	1	**1**
Protozoal cysts skeletal muscle	/	2	7	5	**14**
Polydactylia	/	/	/	1	**1**
Central nervous system, eyes, and ears					
Leptomeningeal hemorrhages	/	/	1	2	**3**
Endophthalmitis	/	/	/	1	**1**
with retinal atrophy	/	/	/	1	**1**
Ectoparasites (ticks)	/	/	2	5	**7**
Hematopoetic and endocrine system					
Thyroid follicular cysts	/	1	3	1	**5**
Follicular hyperplasia (tonsil)	/	/	/	3	**3**
Follicular Hyperplasia (lymph node)	/	/	3	3	**6**
Follicular hyalinosis and depletion (lymph node)	2	1	8	10	**21**
Hemosiderosis lymph node	1	1	/	3	**5**
Anthracosis lymph node	1	/	/	2	**3**
Lymphoid depletion	/	/	/	1	**1**
Adrenalitis	1	2	3	4	**10**
Nodular hyperplasia adrenal gland	/	/	/	2	**2**
Fibrosis and mineralization adrenal gland	1	/	/	/	**1**
Periadrenal bleeding	/	/	1	/	**1**
Follicular hyperplasia (spleen)	/	/	1	/	**1**
Follicular hyalinosis and depletion (spleen)	/	2	11	7	**20**
Follicular hyalinosis (Peyer’s patches)	/	/	1	/	**1**

**Table 4:** Microbiological results from the otters found dead in Schleswig-Holstein between 2015–2020.

Bacteria	Liver	Spleen	Kidney	Lung	Pulm. Lymph Node	Intestine	Mes. Lymph Node	Brain	Repro	Sceletal Muscle	Total
*Acinetobacter* spp.	/	/	/	2	/	/	/	/	/	/	2
*α-haem. Streptococci*	3	2	4	7	2	3	1	1	/	1	24
*Buttiauxella* spp.	/	/	/	1	/	/	/	2	/	/	3
*Candida famata*	/	1	/	/	/	/	/	/	/	/	1
*Carnobacterium divergens*	4	5	6	6	2	2	1	/	/	1	27
*Carnobacterium maltaromaticum*	2	1	1	1	/	1	2	1	1	/	10
*Clostridium colicanis*	/	/	/	/	/	1	/	/	/	/	1
*Clostridium perfringens*	/	/	/	/	/	3	/	/	/	/	3
*Enterococcus faecalis*	/	/	/	1	/	1	/	/	/	1	3
*Erwinia* spp.	/	/	/	/	/	/	/	/	/	1	1
*Escherichia coli*	/	/	/	2	/	1	/	1	/	/	4
*Ewingella americana*	/	/	/	1	1	/	/	/	/	/	2
Yeasts (not specified further)	1	1	1	/	/	/	/	/	/	/	3
*Kurtia* spp.	1	/	1	/	/	/	/	/	/	/	2
*Lactobacillus sakei*	/	/	/	/	/	/	1	/	/	/	1
*Macrococcus caseolyticus*	/	/	/	1	/	/	/	/	/	/	1
*Macrococcus* spp.	/	/	1	1	/	/	1	/	/	/	3
*Micrococcus luteus*	/	/	/	1	/	/	/	/	/	/	1
*Pantoea* spp.	1	2	1	3	/	/	/	/	/	1	8
*Paraclostridium bifermentans*	/	/	/	/	/	1	/	/	/	/	1
*Pseudomonas* spp.	3	2	5	7	3	3	2	1	1	/	27
*Psychrobacter* spp.	2	2	1	2	/	/	/	/	/	/	7
*Rhanella aquatilis*	/	/	/	2	/	/	/	/	/	/	2
*Rhanella* spp.	/	/	1	1	/	/	/	1	/	/	3
*Serratia fonticola*	/	/	/	3	1	/	/	1	/	/	5
*Serratia* spp.	/	/	/	/	/	/	/	1	/	/	1
*Staphylococcus equorum*	/	/	/	1	/	/	/	/	/	/	1
*Staphylococcus lutrae*	/	/	/	/	/	1	/	/	/	/	1
*Staphylococcus saprophyticus*	/	/	/	1	/	/	/	/	/	/	1
*Staphylococcus sciuri*	/	/	/	1	/	/	/	/	/	/	1
*Stenotrophomonas maltophilia*	/	/	/	1	/	/	/	/	/	/	1
*Stenotrophomonas* spp.	/	/	/	/	/	/	/	1	/	/	1
*Streptococcus canis*	/	/	/	1	/	/	/	/	/	/	1
*Streptococcus dysgalactiae*	/	/	/	/	1	/	/	/	/	/	1
*Vagococcus lutrae*	/	/	/	/	/	1	1	/	/	/	2
*Yarrowia lipolytica*	/	/	/	/	/	/	/	/	1	/	1
*γ-haem. Streptococci*	/	/	1	6	1	1	/	2	/	/	11
Total	17	16	23	53	11	19	9	12	3	5	

**Table 5:** Causes of death of 92 Eurasian otters found in Schleswig-Holstein between 2015–2020.

Years of Finding	Cause of Death			
	Trauma	Starvation	Disease	Unclear
unknown years	3	/	/	3
2015–2018	17	/	/	10
2019	26	1	1	/
2020	28	2	/	1
Total	74	3	1	14

## Supplementary Materials

The following are available online at https://www.mdpi.com/article/10.3390/ani12010059/s1. Figure S1: Dissection Protocol-Otter.
