# Supplementary material for: Pathological Findings in Eurasian Otters (Lutra lutra) Found Dead between 2015−2020 in Schleswig-Holstein, Germany
Source: Animals (Basel). 2021 Dec 28;12(1):59. doi: 10.3390/ani12010059 (PMC8749874; doi:10.3390/ani12010059)
Supplement: Supplementary file 1 [file animals-12-00059-s001.zip › animals-1524996-supplementary.pdf]

DISSECTION PROTOCOL – OTTER ***Dissection team:***

Diss.Nr. .... **Spec.** ....

Total length .... cm

Weight .... kg

Date of finding .... Dissection date ....

Sex m w

Location of finding ....

Estimated age: [years] pup/juv. ☐ subadult ☐ adult ☐

Preparation: fresh ☐ frozen ☐

Decomposition grade: ☐ (1-5)

Bone density: yes ☐ no ☐

Chip number:

<u>Preliminary report</u>

Previous number:

Photos
yes ☐
no ☐

Lesions or abnormalities should be recorded in the layout

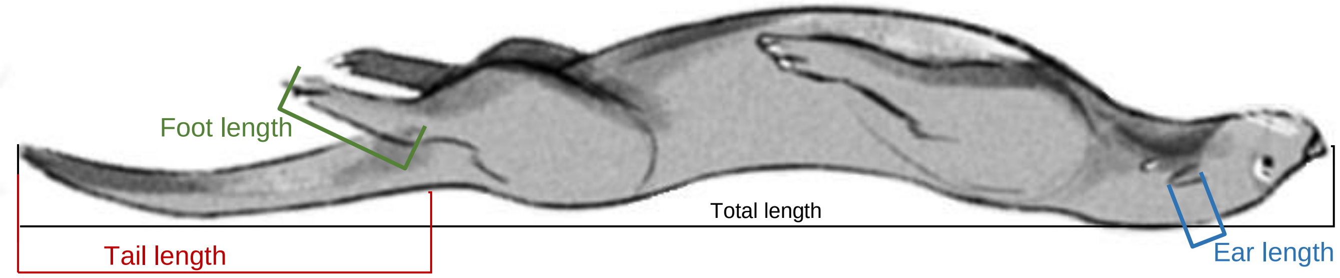

Measured sections: [cm]

(1) .... Total length (Snout tip to tail tip)

(2) .... Head-torso (Snout tip to anus)

(3) .... Tail length (Tail tip to anus)

(4) .... Hind foot length (plantar, without claws)

(5) .... Ear length

(Axillary) Girth .... cm

Mammary glands [mm] **left** upper ……………. **right** upper ……………..

lower ……………. lower ……………..

Fighting injuries/ bitemarks (Eyes, perineum, feet)**:**

Status feet and claws:

Rate of wear teeth:

STIFTUNG · TIERÄRZTLICHE HOCHSCHULE HANNOVER 1778

Diss.Nr. L.l.......................

Nutritional status: good ☐ moderate ☐ poor ☐
Musculature: good ☐ moderate ☐ poor ☐
Fat: good ☐ moderate ☐ poor ☐

Organ weights: [g]

Thyroid gland	le …………		ri ……………
Heart (rinse first!)	....	Spleen	....
Liver	....	Brain	..…………….
Kidneys	le		ri.....................
Adrenal glands	le		ri ....
Testicle incl. Epid.	le …………..		ri ....
Testicle excl. Epid.	le		ri ....
Ovaries	le		ri ….…...

Measurements Reproductive organs:

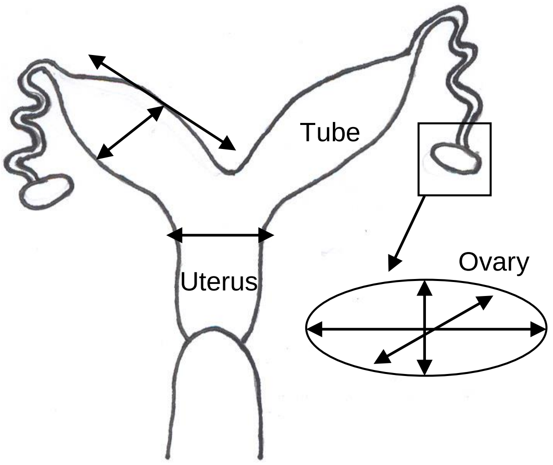

Ovary (ri): L………….. W .... H .... [cm]

Ovary (le): L…………... W ……………. H ………… [cm]

Uterus: .... [cm] (Diameter; change to bifurcation)

Uterine tubes: Ø [mm] le ………….. ri ………………

Length [cm] le ………….. ri ………………

Placental scars [cm]: le ………….. ri ………….. None ☐

Baculum length [cm]:

Testicle + Epid. (ri): L .... W .... H .... [cm]

Testicle + Epid. (le): L .... W ….......…… H ……....… [cm]

Testicle (ri): L .... W .... H .... [cm]

Testicle (le): L .... W .... H .... [cm]

Spermatic duct cysts: quant. right: ….. / space below testicle: ……..[cm] / size [mm] ………

None ☐ quant. left: ..….. / space below testicle: ……..[cm] / size [mm] ………

Urinary bladder: filled ☐ empty ☐ urine saved ☐

Parasites

Lung none ☐ mild ☐ mod. ☐ sev. ☐ location:

Heart none ☐ mild ☐ mod. ☐ sev. ☐ location:

Stomach none ☐ mild ☐ mod. ☐ sev. ☐ location:

Intestine none ☐ mild ☐ mod. ☐ sev. ☐ location:

Liver none ☐ mild ☐ mod. ☐ sev. ☐ location:

Fur (lice comb!) none ☐ mild ☐ mod. ☐ sev. ☐ location:

Renal calculi:

STIFTUNG TIERÄRZTLICHE HOCHSCHULE HANNOVER 1778

Diss.Nr. L.l......................

Sampling for the otter monitoring

**Age determination (Kautex-Bottle with H2O)**		**Virology (Plastic bags, doubled; -70°C)**	
	Teeth in alveolae (Caninus lower jaw)		Lung + lung morbilli
**Parasites (70% Alcohol)**			Tracheal swab
	Lung		Spleen
	Stomach		Liver
	Intestine		Kidney
	Liver		Intestine (2x small, 1x large intestine) + int. parvo
	Heart		Mesent. lymphn.
	Fur		Thymus
			Tonsils
**Histo (Container, formalin 10%; room temp.)**			Brain
	Tongue	**Bacteriology (Plastic bag, -20°; swabs +4°C)**	
	Tonsils		Lung, pulm. lymphn.
	Retroph.lymphn.		Spleen
	Trachea with Larynx		Liver
	Oesophagus		Kidney
	Thyroid gland		Intestine (2x small, 1x large intestine)
	Thymus		Mesent. lymphn.
	Diaphragm		Brain
	Lung (6x: both lungs 1x cran, med, caud, respect.)		Reproduction
	Pulm. lymphn. (teabag)		Swabs
	Heart (with papillary muscle)	**Parasitology (-20°C)**	
	Aorta		gall bladder (bag)
	Liver (6x) + gall bladder		intestinal loops CLOSED (bag)
	Pancreas		muscle, tongue, heart, diaphragm (bag)
	Stomach		faeces (special faeces container)
	Spleen		blood Toxoplasma (serum tube)
	Kidneys (both)	**Toxicology (1x Pl. bag, 1x alu foil; -20°C)**	
	Adrenal glands ri + le (2 teabags)		Rest of the liver (1x bag, 1x Alu)
	Intestine (4 locations)		Kidneys (1x bag, 1x Alu)
	Mesent. lymphn. (teabag)		Muscle (1x bag, 1x Alu)
	Urinary bladder		Fat (1x bag, 1x Alu)
	Ovaries + uterus/ Testicles + ducts (sep. container)		Skin with fur (1x bag, 1x Alu)
	Mammary gland	**Bundesanstalt f. Gewässerkunde (-70°C)**	
	Eye (le)		10-20g liver from all lobes (Alu)
	Muscle	**Pharmacology (-70°C)**	
	Bone marrow (rib)		Brain (2 punches, in 2 Eppendorf tubes)
	Skin + fat	**Genetics (Plastic bag; -20°C)**	
	Brain (one half)		Skin with fur/ muscle (Radix of tail)
	Spinal cord	**Bones (Plastic bag; -20°C)**	
			Humerus and first 3 lumbar vertebrae (DXA) (bag)
			Baculum defleshed (bag)
			5th le rib defleshed (bag)
**Dietary analysis (Plastic bag; -20°C)**		**Additional (Plastic bag; -20°C)**	
	Stomach only hard pads		Eye (ri) (bag)
	Intestinal content		Urine in container
	Muscle		Brain (bag)
**Microplastic analysis (Glas jar, alu-foil + pl. bag; -20°C)**			
	caudal 10cm of Rectum (Glas jar)		Blood **(-70°C)**
	Muscle (first alu-foil, then plastic bag)		**Dry** renal calculi in container with paper (do not freeze)

Blood: if carcass is fresh Serum, Heparin and more at one`s best judgement
